# Supplementary material for: Productivity and Community Composition of Low Biomass/High Silica Precipitation Hot Springs: A Possible Window to Earth’s Early Biosphere?
Source: Life (Basel). 2019 Jul 29;9(3):64. doi: 10.3390/life9030064 (PMC6789502; doi:10.3390/life9030064)
Supplement: Supplementary file 1 [file life-09-00064-s001.pdf]

**Supplementary Material**

This section includes:
(1) Images and descriptions for 'The Dryer',
(2) Supplementary Table 1, and
(3) Select EDX results and descriptions for SEM images shown in the main text (Figures 4–10).

## 1. Shifting Geochemistry at 'The Dryer' Results in a Shift in Community Composition

Prior to 2010, 'The Dryer' had a similar appearance (at least as far back as 2003 based on personal observation by the authors), with no built-up phototrophic mat or orange and brown pigments visible. The thick phototrophic mat which formed during the geochemical shift (2010 and 2011) was rapidly silicified, preserving mat textures after the phototrophic mat has lost all pigment (upper right, 2012). As pigmentation present during the mat building was quickly lost (between the 2011 and 2012 image), it is assumed that the community composition also experienced a change.

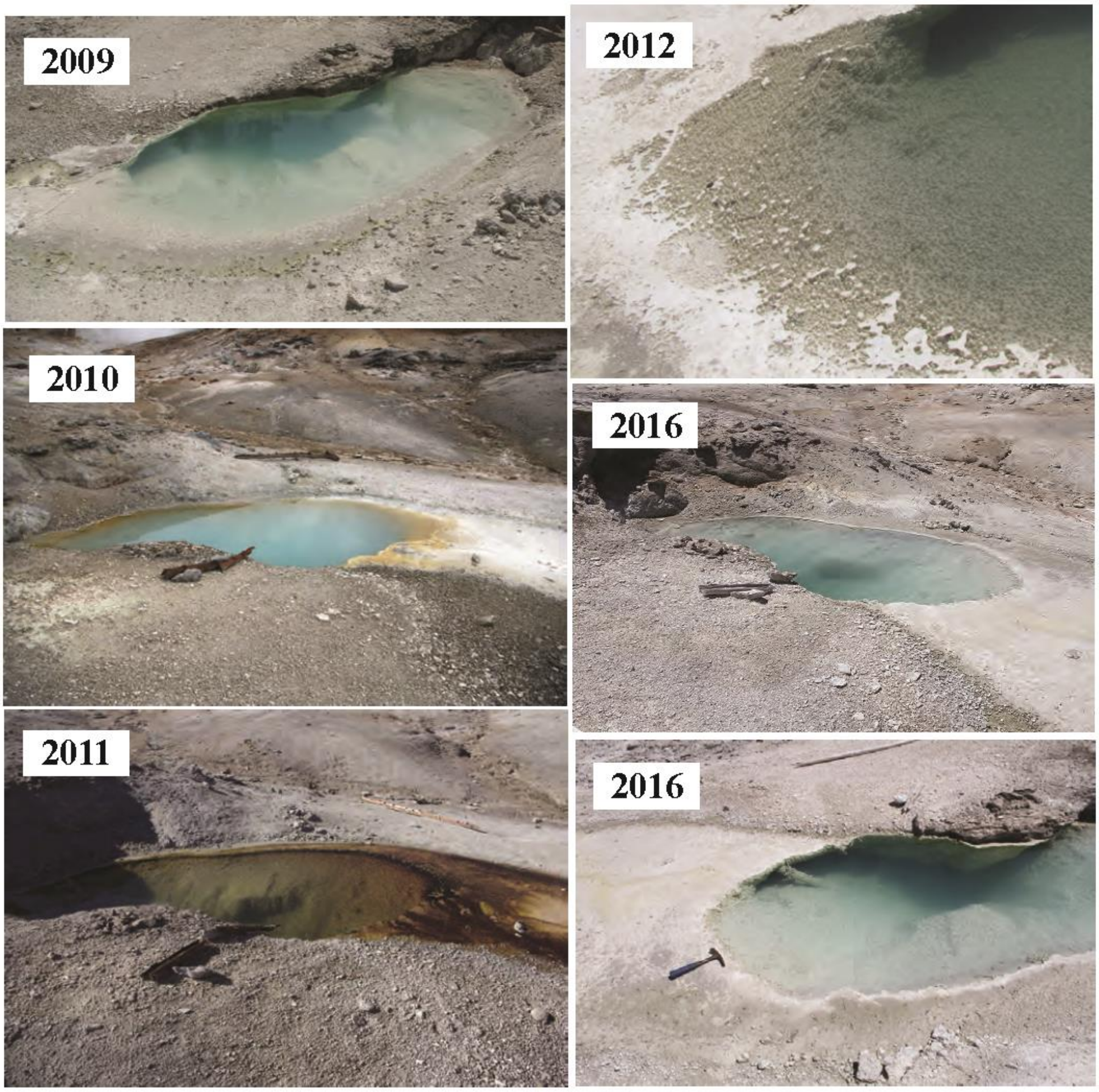


**SOM Figure 1.** Images showing the progression of the development and loss of a phototrophic mat at 'The Dryer', SSA, GGB from 2009 to 2016, highlighting phototrophic mat formation associated with presumed geochemistry shift to higher pH 2010–2011 and then return to pre-2010 geochemistry and lower pH after 2012.

## 2. Carbon Uptake Data Used to Produce Figure 3

**Supplementary Table 1.** Carbon uptake experiment results. All results reported in units of μg C uptake/g Cbiomass/hr.

| Site Name | Date | Treatment | Rep 1 | Rep 2 | Rep 3 | Average | St. Dev. |
|---|---|---|---|---|---|---|---|
| 'Heartbeat Pool' | June, 2018 | Light | 2.08 | 7.42 | 3.97 | 4.49 | 2.71 |
| | | Dark | 6.53 | 5.65 | 6.76 | 6.31 | 0.59 |
| Dante's Inferno | July, 2016 | Light | 21.42 | 20.96 | 21.58 | 21.32 | 0.32 |
| | | Dark | 16.32 | 20.59 | 22.05 | 19.65 | 2.98 |
| 'Avocado Spring' | July, 2016 | Light | 17.69 | 22.15 | 8.83 | 16.22 | 6.78 |
| | (grey mat) | Dark | 3.04 | 3.55 | lost | 3.30 | 0.36 |
| | June, 2018 | Light | 23.41 | 15.49 | 28.79 | 22.56 | 6.69 |
| | (red mat) | Dark | -3.27 | -4.31 | 3.99 | -1.20 | 4.52 |
| 'The Dryer' | July, 2016 | Light | 396.54 | 312.46 | 357.04 | 355.35 | 42.06 |
| | | Dark | 8.71 | 6.15 | 8.86 | 7.91 | 1.53 |
| Boulder Geyser OF | June, 2017 | Light | 24.74 | 16.06 | 20.79 | 20.53 | 4.35 |
| | | Dark | 2.54 | 25.23 | 27.31 | 18.36 | 13.74 |
| 'Rose Terrace Pool' | June, 2018 | Light | 16.09 | 4.99 | 18.26 | 13.11 | 7.12 |
| | | Dark | 9.55 | 18.87 | 20.94 | 16.45 | 6.07 |

Rep = replicate, St. Dev. = standard deviation calculated from the replicates, lost = replicate was lost due to container breaking in transit, OF = outflow.

## 3. Characterization of Materials via SEM-EDX

Spot analyses were conducted via EDX (as described in the methods section) to determine the composition of materials observed in SEM images. The following are examples of analytical results for types of materials highlighted in SEM images: EPS (extra-cellular polymeric substances and associated cellular biomass), silica (mostly amorphous $SiO_2$ with a significant amount of aluminum, and usually associated with microbial EPS/biomass), particulates (typically alumino-silicates and/or silica), sulfur (elemental sulfur), Fe-S (iron sulfide minerals), and Fe-oxides (iron oxides minerals).

***EPS (Biomass)*:** predominantly made up of carbon, oxygen, and nitrogen. This example is from material labelled 'EPS' (extra-cellular polymeric substance) in Figure 8F, from the 'Avocado Spring' mat sample collected in 2018. Due to the drying process, all EPS and biomass has lost most of its mass, and distinguishing between a bacterial cell and the EPS that had coated it is not possibly via these means. As a result, we have lumped all EPS and biomass together into one category.

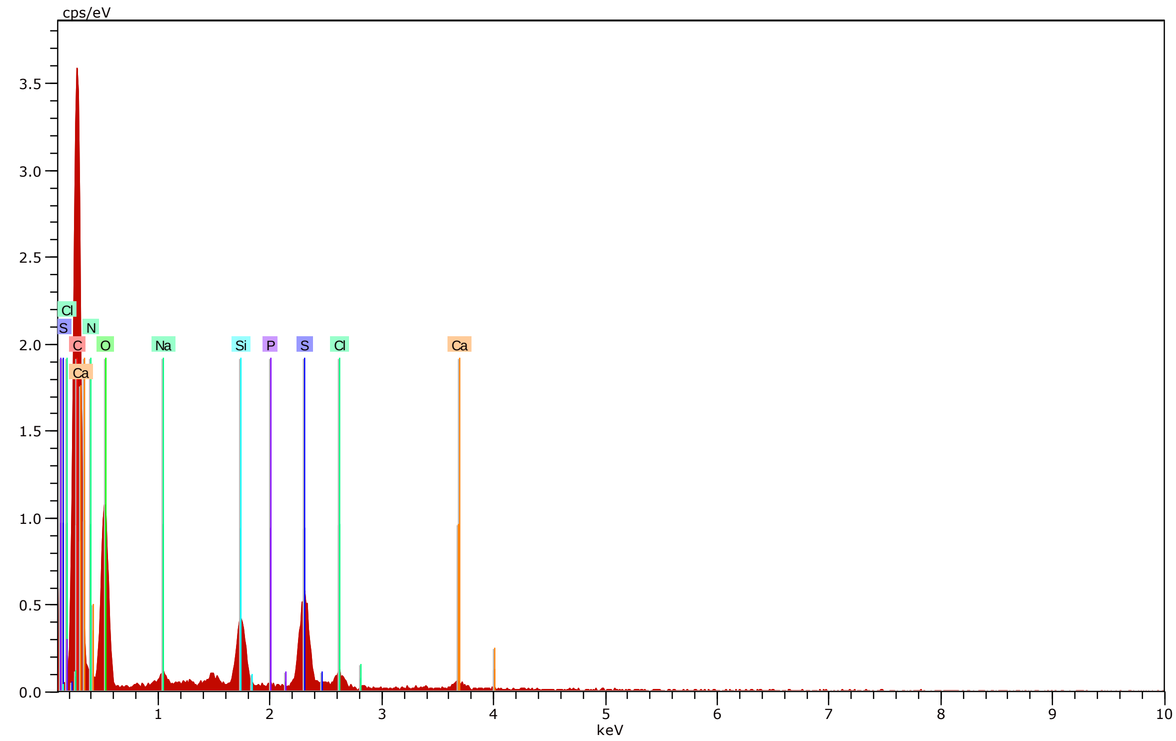


| Element | AN | Series | unn. C [wt.%] | norm. C [wt.%] | Atom. C [at.%] | Error [%] |
|---|---|---|---|---|---|---|
| Carbon | 6 | K-series | 57.06 | 57.06 | 65.00 | 11.0 |
| Oxygen | 8 | K-series | 28.38 | 28.38 | 24.27 | 4.4 |
| Nitrogen | 7 | K-series | 7.91 | 7.91 | 7.73 | 3.3 |
| Sulfur | 16 | K-series | 2.93 | 2.93 | 1.25 | 0.1 |
| Silicon | 14 | K-series | 1.80 | 1.80 | 0.88 | 0.1 |
| Sodium | 11 | K-series | 0.72 | 0.72 | 0.43 | 0.1 |
| Chlorine | 17 | K-series | 0.60 | 0.60 | 0.23 | 0.1 |
| Calcium | 20 | K-series | 0.49 | 0.49 | 0.17 | 0.1 |
| Phosphorus | 15 | K-series | 0.10 | 0.10 | 0.05 | 0.0 |
| | | Total: | 100.00 | 100.00 | 100.00 | |

***Silica (silica containing aluminum)***: Predominantly made up of amorphous silica and oxygen but also containing aluminum. This example is from material labelled ‘Silica’ in Fig 10F, from the ‘The Dryer’ silica sample. Silica is mostly autochthonous, having precipitated from the hydrothermal water, though allochthonous silica precipitate particles can be blown in, and the methods we have used are not sensitive to distinguishing between the two.

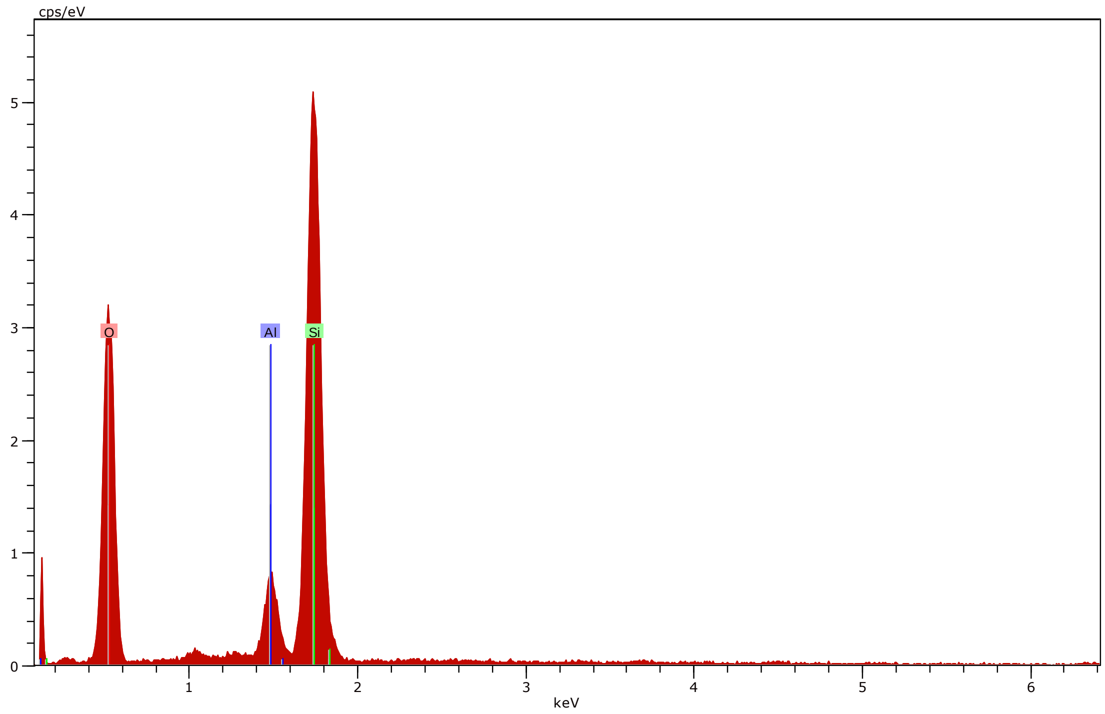


| Element | AN | Series | unn. C [wt.%] | norm. C [wt.%] | Atom. C [at.%] | Error [%] |
|---|---|---|---|---|---|---|
| Oxygen | 8 | K-series | 53.80 | 55.85 | 68.84 | 6.9 |
| Silicon | 14 | K-series | 37.25 | 38.68 | 27.16 | 1.6 |
| Aluminium | 13 | K-series | 5.27 | 5.47 | 4.00 | 0.3 |
| | | Total: | 96.32 | 100.00 | 100.00 | |

***Silica (predominantly silica containing aluminum)***: Predominantly made up of amorphous silica and oxygen containing aluminum, and often co-occurring with carbon (biomass). This example is from material labelled 'EPS' (due to the relatively high carbon content) in Figure 7F, from the Dante's Inferno sediment sample, highlighting the co-occurrence of carbon with silica typical of microbial biomass in hydrothermal settings as has been observed previously (e.g., [24]).

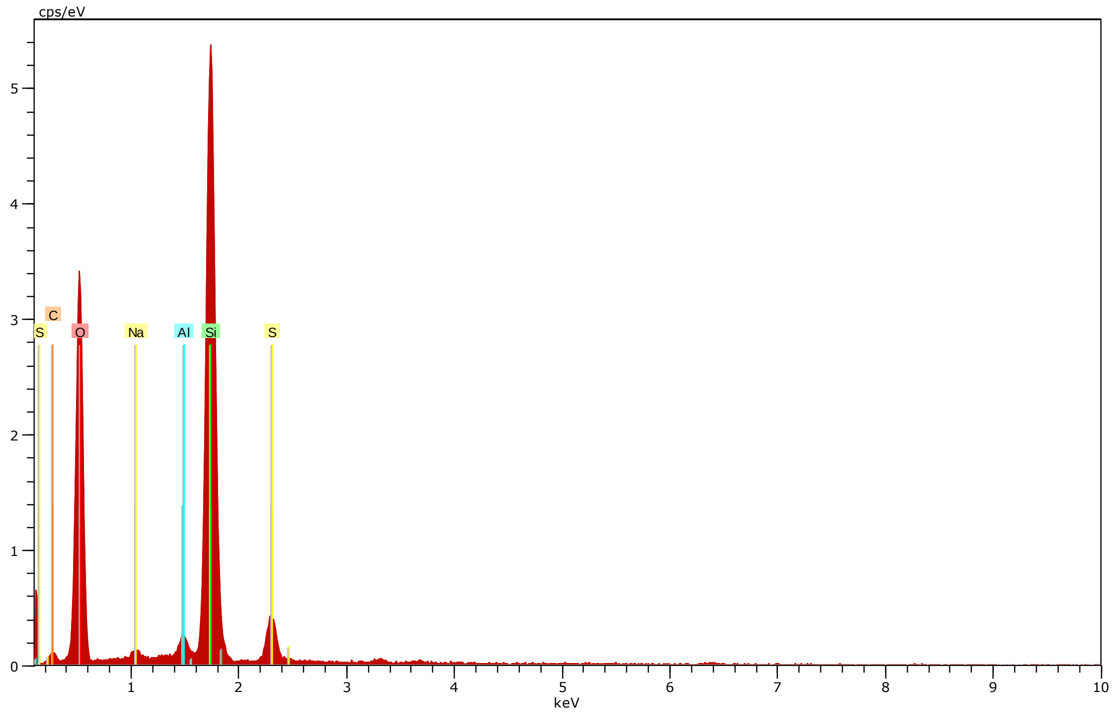


| Element | AN | Series | unn. C [wt.%] | norm. C [wt.%] | Atom. C [at.%] | Error [%] |
|---|---|---|---|---|---|---|
| Oxygen | 8 | K-series | 63.95 | 54.43 | 62.85 | 8.1 |
| Silicon | 14 | K-series | 37.13 | 31.60 | 20.79 | 1.6 |
| Carbon | 6 | K-series | 9.68 | 8.24 | 12.67 | 2.3 |
| Sulfur | 16 | K-series | 3.90 | 3.32 | 1.91 | 0.2 |
| Aluminium | 13 | K-series | 1.62 | 1.38 | 0.94 | 0.1 |
| Sodium | 11 | K-series | 1.22 | 1.03 | 0.83 | 0.1 |
| | | Total: | 117.49 | 100.00 | 100.00 | |

***Particulate***: Alumino-silicate - this example is from material labelled 'Part.' in Figure 8E, from the 'Avocado Spring' mat sample collected in 2018. This most likely is of a piece of local rock (rhyolitic tuffs, glasses, and rock that has been ground to find particles by glaciers and erosion) given the relatively high Al, Na, and K content, suggesting rock that has not lost a lot of cations through chemical weathering.

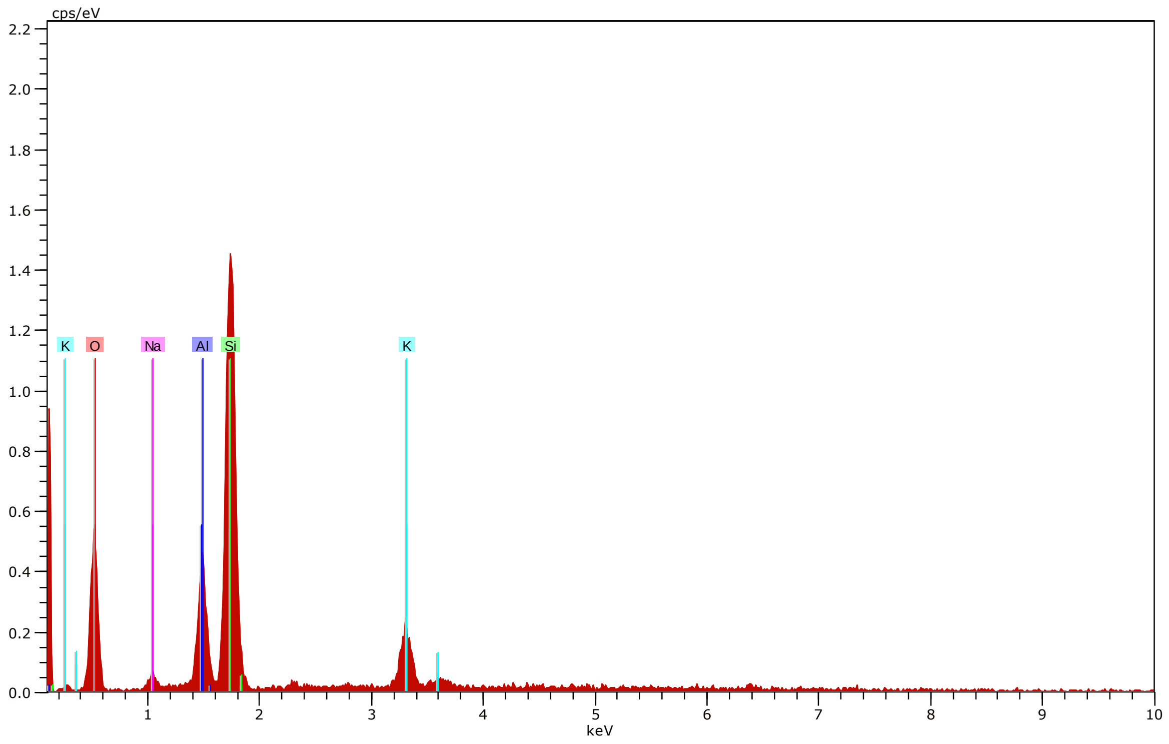


| Element | AN | Series | unn. C [wt.%] | norm. C [wt.%] | Atom. C [at.%] | Error [%] |
|---|---|---|---|---|---|---|
| Silicon | 14 | K-series | 62.49 | 41.90 | 32.03 | 2.8 |
| Oxygen | 8 | K-series | 60.52 | 40.58 | 54.46 | 11.0 |
| Aluminium | 13 | K-series | 17.43 | 11.69 | 9.30 | 1.0 |
| Potassium | 19 | K-series | 4.81 | 3.22 | 1.77 | 0.2 |
| Sodium | 11 | K-series | 3.89 | 2.61 | 2.44 | 0.4 |
| | | Total: | 149.14 | 100.00 | 100.00 | |

***(Elemental) Sulfur***: Predominantly made up of sulfur, though often with minor amounts of other elements (e.g., C, Si, O) due to the co-occurrence of biofilms (EPS, biomass) and ubiquitous silica (silica-saturated hydrothermal water). This example is from material labelled 'Sulfur' in Figure 7C, from the Dante's Inferno sediment sample. All sulfur samples observed from Dante's Inferno exhibit a coating of organic material with associated silica, indicating the presence of a microbial community at the time of collection. The surfaces are also all mottled with pits and valleys that are roughly similar in diameter to microbial cells, and there is a correlation between loss of size and spherical shape and increasing thickness of EPS/biomass. All of this circumstantial evidence suggests the sulfur spheres are colonized and oxidized by endemic microbial communities.

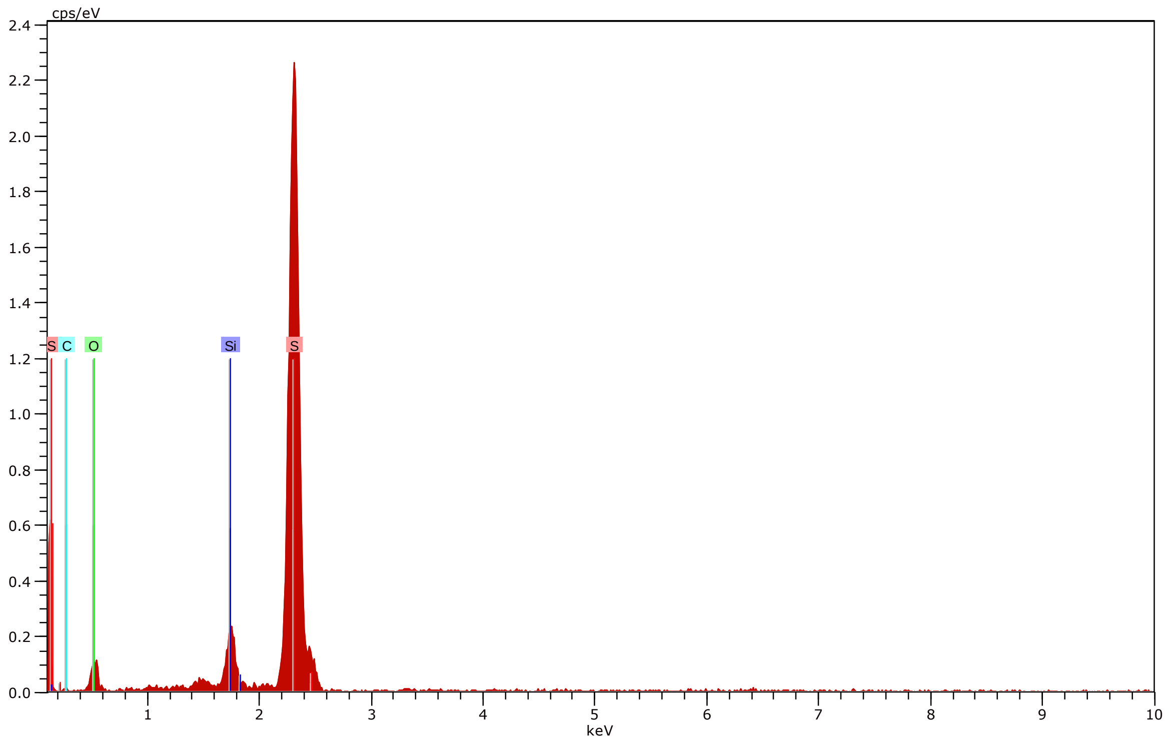


| Element | AN | Series | unn. C [wt.%] | norm. C [wt.%] | Atom. C [at.%] | Error [%] |
|---|---|---|---|---|---|---|
| Sulfur | 16 | K-series | 71.29 | 69.94 | 53.93 | 2.6 |
| Oxygen | 8 | K-series | 17.76 | 17.42 | 26.93 | 4.7 |
| Carbon | 6 | K-series | 6.94 | 6.81 | 14.02 | 4.5 |
| Silicon | 14 | K-series | 5.93 | 5.82 | 5.12 | 0.4 |
| | | Total: | 101.92 | 100.00 | 100.00 | |

***Iron sulfide minerals (Fe-S):*** Predominantly made up of sulfur and iron, though often with minor amounts of other elements (e.g., C, Si, O) due to the co-occurrence of biofilms (EPS, biomass) and ubiquitous silica (silica-saturated hydrothermal water). This example is from material labelled 'Fe-S' in Figure 7E, from the Dante's Inferno sediment sample. Due the age of the instrument, we are unable to confidently characterize the Fe-S mineralogy based on Fe:S ratios.

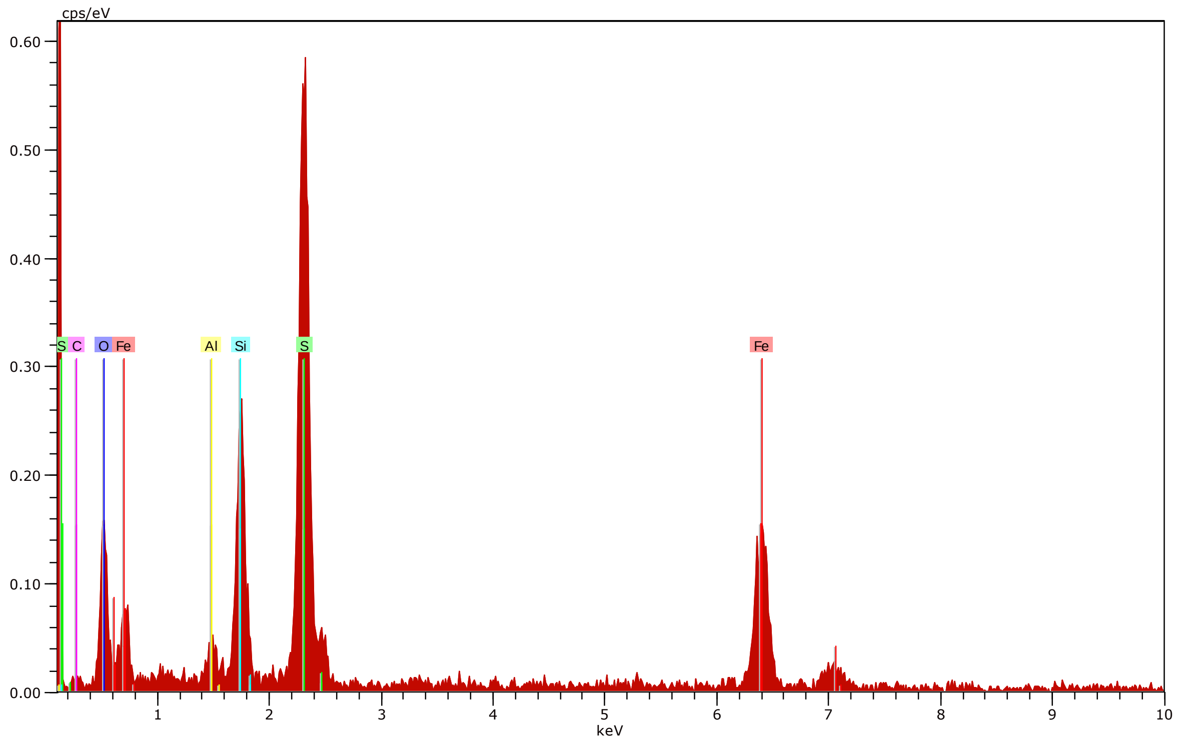


| Element | AN | Series | unn. C [wt.%] | norm. C [wt.%] | Atom. C [at.%] | Error [%] |
|---|---|---|---|---|---|---|
| Iron | 26 | K-series | 42.57 | 39.29 | 20.57 | 1.8 |
| Sulfur | 16 | K-series | 29.31 | 27.05 | 24.66 | 1.2 |
| Oxygen | 8 | K-series | 17.14 | 15.82 | 28.91 | 4.4 |
| Silicon | 14 | K-series | 11.65 | 10.75 | 11.19 | 0.7 |
| Carbon | 6 | K-series | 5.60 | 5.17 | 12.59 | 3.6 |
| Aluminium | 13 | K-series | 2.08 | 1.92 | 2.08 | 0.2 |
| | | Total: | 108.34 | 100.00 | 100.00 | |

***Iron oxide minerals (Fe-oxides):*** Predominantly made up of oxygen and iron, though often with other elements (e.g., Si, Al) due to the co-occurrence of ubiquitous aluminum-containing silica sinter (silica-saturated hydrothermal water) and minor amounts of other elements (e.g., Na and Cl precipitated due to drying of the sample). This example is from material labelled 'Fe-oxides' in Fig 6F, from the 'Rose Terrace Pool' sediment sample. The complete lack of any sulfur is the reason these minerals are categorized as Fe-oxides.

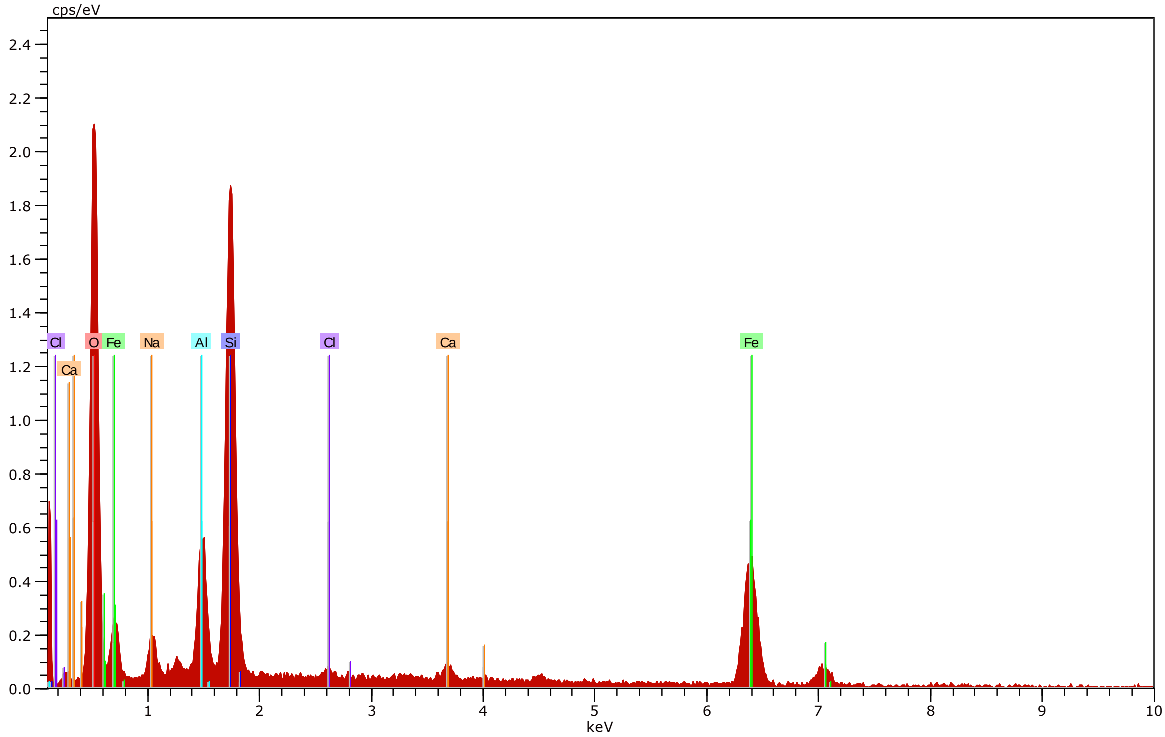


| Element | AN | Series | unn. C [wt.%] | norm. C [wt.%] | Atom. C [at.%] | Error [%] |
|---|---|---|---|---|---|---|
| Oxygen | 8 | K-series | 40.00 | 39.84 | 60.70 | 5.5 |
| Iron | 26 | K-series | 31.04 | 30.91 | 13.49 | 1.0 |
| Silicon | 14 | K-series | 19.07 | 18.99 | 16.48 | 0.8 |
| Aluminium | 13 | K-series | 5.65 | 5.62 | 5.08 | 0.3 |
| Sodium | 11 | K-series | 3.03 | 3.02 | 3.20 | 0.3 |
| Calcium | 20 | K-series | 0.84 | 0.83 | 0.51 | 0.1 |
| Chlorine | 17 | K-series | 0.78 | 0.78 | 0.53 | 0.1 |
| | | Total: | 100.41 | 100.00 | 100.00 | |
